# Editorial: Biology of Ly-6 Supergene Family in Health and Disease

**DOI:** 10.3389/fcell.2022.949379

**Published:** 2022-06-23

**Authors:** Anil K. Bamezai, Julie M. Miwa

**Affiliations:** ^1^ Department of Biology, Villanova University, Villanova, PA, United States; ^2^ Department of Biological Sciences, Lehigh University, Bethlehem, PA, United States

**Keywords:** Ly-6, LU domain, Ly6/uPAR, cell signaling, Ly6 proteins

## Historical Perspective

Ly-6 (or Ly6) proteins were discovered in the mid-1970s and first described as differentially expressed alloantigens on mouse immune cells (Mckenzie et al., 1977; Woody, 1977). The biochemical characterization of Ly-6 proteins, cloning cDNA and chromosomal DNA sequences regulating their expression were reported in the 1980s (reviewed in Rock et al., 1989; Bamezai 2004). Generation of monoclonal antibodies against Ly-6 proteins in mid-1980s and early1990s aided in in-depth investigations into their constitutive and cytokine (e.g., type 1/2 interferons) regulated cellular expression, biochemical characterization as well as provided insights in their possible physiological role (reviewed in Rock et al., 1989; Bamezai 2004). Collectively these reports show that Ly-6 glycoproteins are either secreted or membrane anchored through glycosyl-phosphatidyl-inositol (GPI) lipid moiety, 12–14 kda, cysteine-rich glycoproteins. Role of Ly-6 proteins in cell adhesion and as regulators of signal transduction in immune (e.g., T lymphocytes) (Stanford et al., 1997; Henderson et al., 2002), and non-immune (e.g., neurons) cells (Miwa et al., 1999) has come to light. Besides immune (lymphocytes, granulocytes, macrophages), the non-immune cells (neurons, epithelial cells, gametes) across many species, such as worms, flies, mice, and humans exhibit expression of Ly-6 protein(s). Interestingly, all secreted or membrane anchored Ly-6 proteins show structural resemblance to the three-fingered venom toxins and urokinase Plasminogen Activating Receptor (uPAR) and share one or more “Ly-6/uPAR (LU) domain” (Tsetlin, 1999).

## Summary of Frontier contributions

The invited original and review articles in this volume report recent discoveries aiding in better understanding of the biology of LU proteins. Kulbastskii et al., show colocalization of endogenous GPI-tethered Lypd6 with α3β4-and α7-nAChRs in primary cortical and hippocampal neurons. These authors also report Lypd6 as the endogenous negative modulator of the cholinergic system in the brain. Bychkov et al., discuss SLURP-1 as a secreted LU-related protein-1 that negatively regulates nicotinic receptor α-7nAChR in controlling growth of lung adenocarcinoma cell line by downregulating a cell survival/metabolic pathway associated with PI3K/AKT/mTOR. This report shows, in addition to interaction with α-7nAChR, SLURP-1 binds to platelet-derived growth factor receptor type α (PDGFRβ) and epidermal growth factor receptor (EGFR). Furthermore, their data highlights the role of SLURP-1’s “loop I” in binding to α7-nAChR and altering cell proliferative response with druggable synthetic SLURP-1 loop-1 mimics. Doss et al., report on LYNX1, specifically examining the interaction between LYNX1 and nicotinic acetylcholine receptor (nAChR) subtypes in skeletal neuromuscular junctions (NMJs). The authors’ data support of the role of LYNX1 in maintaining the structure and function of adult and aging NMJs. Kristensen et al., in their review article summarize recent developments in the biology of GPIHBP1, a GPI-anchored LU protein. This member of LU family regulates plasma triglyceride metabolism by interacting and functionally charging the Lipoprotein lipase (LPL) enzyme. These authors discuss the role of GPIHBP1’s intrinsically disordered, highly acidic N-terminal extension and the folded disulfide bond–rich three-fingered LU domain in ushering LPL for intravascular lipolysis. In another review article, Leth and Ploug, discuss recent development in the role of urokinase plasminogen activation receptor, known to bind uPA and regulating uPA’s extravesicular serine protease activity for fibrinolysis, cell adhesion and migration. Authors highlight the upregulated uPAR protein expression at the boundary between the solid tumor and the normal tissue allowing for recognition and complete recitation (surgical/non-surgical) of the tumor tissue. Authors discuss targeting tumors with killer T cells or uPAR-targeted reporter-molecules containing positron emitting radionuclides or near-infrared (NIR) florescence probes. These probes allow tumor detection with tomography (PET) and/or its removal after illuminating it.

## Perspectives

Over the last 50 years the use of LU proteins as markers of hematopoietic stem cells and their progeny has been vital for identifying and understanding the functional responses generated by a variety of immune cell subsets. The reports in this volume and summarized above highlight the broader tissue expression of LU proteins and their role in myriad biological process. LU proteins are known to function as a signaling rheostat by fine tuning and/or regulating signals triggered by other membrane receptor as previously reviewed (Bamezai 2004). For example, Ly-6A expression regulates signaling through the antigen receptor in T lymphocytes (Stanford et al., 1997; Henderson et al., 2002), while LYNX1 binds and regulates signaling through acetylcholine receptor (Miwa et al., 1999), the Sleepless protein regulates a potassium ion channel (Shaker) in neurons (Koh et al., 2008). These interactions between the membrane anchored LU protein and other receptors appear to occur either “in-cis”, meaning within the plane of plasma membrane through a cell-autonomous mechanism, or “in-trans” where the secreted LU protein engages its target membrane receptor to drive the autocrine/paracrine cellular signaling. Additionally, the reported ligand(s) engaging GPI-anchored LU protein may contribute indirectly by regulating signaling in non-cell autonomous manner “in-trans”. Putative soluble or cell-bound ligands directly interacting with LU proteins have been reported (English et al., 2000; Plugh et al., 2002); however, much needs to be done to fully characterize ligands for some Ly-6 family members and to fully understand the signal transduction through these tail-less, GPI-anchored proteins in the absence of their transmembrane and cytoplasmic domains. Contribution of cholesterol-rich lipid rafts on the membrane in signaling through LU proteins has been observed (Lang et al., 2017) and is currently under investigation. LU-proteins are housed in these membrane nanodomains because of their lipid anchor to allow for their interaction and regulation of transmembrane receptors/ion channels residing in lipid rafts (Koh et al., 2008). Another emerging area of investigation is the use of some LU-domain proteins as markers of tumors and as possible targets of either antibody directed and/or cell-based immune therapies (reviewed in Upadhyay, 2019).
